# Epidemiological, clinical and diagnostic profile of breast cancer patients treated at Potchefstroom regional hospital, South Africa, 2012-2018: an open-cohort study

**DOI:** 10.11604/pamj.2020.36.9.21180

**Published:** 2020-05-08

**Authors:** Baudouin Kongolo Kakudji, Prince Kasongo Mwila, Johanita Riétte Burger, Jesslee Melinda Du Plessis

**Affiliations:** 1 Potchefstroom Hospital, Potchefstroom, North West Province, South Africa; 2 Department of Surgery, School of Clinical Medicine, Faculty of Health Sciences, University of the Witwatersrand, Johannesburg, South Africa; 3 Medicine Usage in South Africa (MUSA), Faculty of Health Sciences, North-West University, Potchefstroom, South Africa

**Keywords:** Breast cancer, epidemiology, clinical profile, upper-middle-income country, Potchefstroom, South Africa

## Abstract

**Introduction:**

Breast cancer is the second most diagnosed cancer worldwide. We aimed to depict the diagnostic approach as well as the epidemiological and clinical profile of patients with breast cancer at Potchefstroom regional hospital, South Africa.

**Methods:**

This descriptive open-cohort study included patients with primary invasive breast cancer, confirmed by histology results and treated at the hospital from 01 January 2012 to 31 December 2018. Data such as demographics, patient history, histology, breast clinical findings, physical mass description and diagnostic investigations were captured from hospital registries and patient files.

**Result:**

One-hundred thirty-eight patients (mean age 56.2 (SD: 14.4) (95% CI 54.6-59.7) years) met inclusion criteria. Most patients were female (98.6%), from African (67.4%) or Caucasian (23.9%) descent. Findings included mostly left-sided breast involvement (51.8%), lesions in the upper-outer quadrant (43.1%), extensions to the skin (25.6%, N = 39), and tumour size of 2 ≤ 5 cm (49.3%), or > 5 cm (39.1%). Most patients (57.9%, N = 135) were categorised as BIRADS-5, with a ductal pattern (89.6%) (p < 0.01). Patients mostly presented in stages II to IV of disease (89.1%; p < 0.05). Late-stage (stages III-IV) at time of diagnosis (n = 84) was significantly associated with mass location (p = 0.006; Cramér's V = 0.280), tumour size (p < 0.001, Cramér's V = 0.239), and skin changes (p = 0.027, Cramér's V = 0.492).

**Conclusion:**

Most patients consulted at a late-stage of the disease, indicating a need for the promotion of breast awareness campaigns, early detection, and timeous referral.

## Introduction

Breast cancer is the second most diagnosed cancer accounting for an estimated 2.1 million cases (11.6%) of all diagnosed cancers worldwide, and a leading cause of cancer-related morbidity and mortality with more than 626 000 deaths in 2018 [1]. Based on statistics from the South African National Cancer Registry, 8 230 new breast cancer patients were diagnosed in 2014, representing 21.8% of all cancers in the country at the time [2]. The World Bank [3] classifies countries in four groups according to their Gross National Income (GNI) per capita, as low-income, lower-middle-, upper-middle- and high-income countries. Although South Africa is formally classified as an upper-middle-income country [3]; it has been described as “both developed and under-developed at the same time, being home to cosmopolitan city centres, comfortable neighbourhoods and suburbs, but also to impoverished townships” [4]. The country is divided into nine provinces, which vary considerably in size. Gauteng, a highly urbanised region is the smallest, whereas the North West Province is the 6th largest in terms of size. The population of the North West Province resemble that of a developing country with approximately 60% of the population living in rural areas [5]. In lower- to middle-income countries, breast cancer patients often present late in their diagnostic stage with advanced disease [6-8]. The clinical stage at which patients present when diagnosed with breast cancer is a vital prognostic survival factor that may challenge the management thereof [9]. Cancer is usually detected during a screening mammogram, where patients with Breast, Imaging, Reporting and Data System (BIRADS) four and five will have a biopsy of the breast lesion done. BIRADS is a lexicon that forms part of a standardised mammography report whereby findings are put in a small number of well-defined categories that aim to aid quality assurance, communication, research, and improved patient care [10]. The screening mammogram and the greater breast cancer awareness of patients allow earlier diagnosis (stage I or II) [10]. The goals of the South African National Department of Health's breast cancer control and management policy [11] include the improvement of survival, decreased time to presentation, patient diagnosis and treatment initiation. It furthermore aims to decrease the stage at which treatment is started, thereby improving the quality of life in both survivorship and palliation, and to effectively monitor and evaluate the programme implementation and the impact of breast cancer interventions. Within South Africa itself, some disparities have been noted between regions and population groups in an early study [12]. With the South African National Department of Health's policy goals at hand and the attentiveness of disparities regarding access to healthcare, the need for local/regional profiling of breast cancer patients derived. The aim of the study was to depict the diagnostic approach as well as the epidemiological and clinical profile of breast cancer in Potchefstroom regional hospital, South Africa. The study is, therefore, a snapshot of the current status in a rural area and should pave the way for improvement.

## Methods

**Study population and setting:** a retrospective, observational open-cohort study was conducted between February and July 2019 that included data from 01 January 2012 to 31 December 2018. Data were obtained from hospital registers (breast and surgical clinic registers, ward registers, theatre register) and patient files. All patients with primary invasive breast cancer that were confirmed by histology results, treated at the Potchefstroom Hospital, North West Province, South Africa, was included and resulted in a total of 138 patients that met the inclusion criteria. Patients with incomplete data fields (missing histology) and breast tumours from another origin (breast metastasis from another primary site than breast tissue) were excluded. Potchefstroom Hospital is strategically located a 109 km from Chris Hani Baragwanath Hospital (central academic hospital) in Johannesburg with its world-class academic health facilities and approximately 50 km from the North West Province tertiary hospital (Klerksdorp/Tshepong Hospital complex) with an oncology department. Potchefstroom Hospital is a newly established regional hospital and currently has a surgical team which is very involved in breast cancer management. It furthermore has a breast clinic that has been functioning for more than five years. Specimens for histology are processed by the National Health Laboratory Service and mammograms are done at Tshepong Hospital whereby results are then sent to Potchefstroom Hospital. Oncology treatment is initiated and monitored in Klerksdorp Hospital, while patient optimisation and follow-up are done in Potchefstroom Hospital.

**Data collection:** a data collection tool, consisting of a Microsoft Word® document, was developed using the different data fields available in the hospital registers and patient files. Data fields for the study included the following: date of surgery, date of the first consultation, histology information, demographic information (i.e. date of birth, gender and population group), patient history, breast clinical findings, physical mass description, breast diagnostic investigation findings and patient's human immunodeficiency virus/acquired immunodeficiency syndrome (HIV/AIDS) status. The tool was converted to a Microsoft Excel® data capturing sheet. A 5% data re-entry method was followed, whereby 5% of the data were entered into a different dataset, where after the datasets were compared electronically. All discrepancies flagged as errors were resolved manually by comparing the electronic dataset to the data collection tool, using the patient number indicated on the tool/sheet. If any discrepancies were found, another 5% of data were re-entered and the process repeated until no discrepancies were found. Data were also checked for any outliers. Age group was divided into 10-year intervals. Population group was categorised as either African (urban and non-urban), Indian, Coloured, Caucasian and other as demarcated in the South African Demographic and Health Survey [5].

**Statistical analysis:** data were analysed using SPSS^®^ version 25. Tests for normality (Q-Q plots) were used to determine data distribution. Continuous variables were expressed as means, standard deviations and 95% confidence intervals (CIs), and tested for heterogeneity of means by analysis of group variances using the student t-test. Categorical variables were expressed as counts and percentages. To test if single categorical variables follow a hypothesised population distribution, we performed the one-sample chi-square test, whereas Pearson's chi-square test and Fisher's exact test were used for the assessment of the relationship between categorical variables. A two-tailed p-value, where p < 0.05, was considered significant. The practical significance of associations was computed when p-values were significant. To determine the effect size of associations, we used the Cramér's V statistic. A Cramér's V ≥ 0.1 was deemed as a weak association, Cramér's V ≥ 0.3 was seen as a moderate association and Cramér's V ≥ 0.5 regarded as a large effect/association. To assess the size of the difference between means we used Cohen's d-value, with d ≥ 0.8 regarded as practically significant. To describe the association between the demographic, epidemiological and clinical profile of breast cancer patients in Potchefstroom Hospital, we grouped patients as either early stage (ES) (stages I and II) or late-stage (LS) (stages III and IV). Table 1 present the demographic characteristics of the study population. Table 2 and Table 3 show the findings of the clinical examination and physical mass description, and diagnostic investigation respectively. Percentages in each category were calculated according to valid (non-missing) cases as the denominator.

**Table 1:** Demographic characteristics of the study population

	n	%*
**Total number of patients, N**	**138**	**100**
**Gender (N = 138)**		
Male	2	1.5
Female	136	98.6
**Age, groups, years (N = 138)**		
<29	3	2.2
30-39	20	14.5
40-49	29	21.0
50-59	31	22.5
60-69	25	18.2
≥70	30	21.7
**Population group (N = 138)**		
African	93	67.4
Coloured	11	8.0
Indian	1	0.7
Caucasian	33	23.9
**Family history of cancer (N = 18)**		
None	2	11.1
Breast cancer	4	22.2
Ovarian cancer	5	27.8
Prostate cancer	3	16.7
Uterine cancer	4	22.2
**Prior hormone therapy (N = 84)**		
None	56	66.7
Contraceptives	21	25.0
Hormone replacement therapy	7	8.3
**HIV status (N = 122)**		
HIV-negative	89	72.9
HIV-positive	33	27.1

* Percentage calculated using the total number of non-missing values in each category as the denominator

**Table 2:** Clinical examination and physical mass description

	n	%*
**Side affected (N = 137)**		
Left	71	51.8
Right	65	47.5
Bilateral	1	0.7
**Location (N = 137)**		
Upper outer	59	43.1
Upper inner	28	20.4
Lower outer	13	9.5
Lower inner	14	10.2
Retro-areolar	11	8.0
All 4 quadrants	12	8.8
**Tumour size (N = 138)**		
< 2cm	13	9.4
> 2 ≤ 5	68	49.3
> 5cm	54	39.1
Not applicable (Paget's disease)	3	2.2
**Tumour local invasion (N = 39)**		
No fixation	15	38.5
Skin	10	25.6
Deep structures	6	15.4
Skin and deep structure	8	20.5
**Ulceration (N = 31)**		
None	24	77.4
≤ 2 cm	1	3.2
> 2 ≤ 5 cm	3	9.7
> 5 cm	3	9.7
**Discharge (N = 32)**		
None	17	53.1
Watery	3	9.4
Pus/Greenish	3	9.4
Bloody	9	28.1
**Skin changes (N = 39)**		
None	15	38.5
Puckering	6	15.4
Dimpling	3	7.7
Rash/redness	2	5.1
Peau d'orange	11	28.2
Puckering and dimpling/redness	2	5.1
**Nipple changes (N = 26)**		
None	21	76.9
Unspecified skin lesions	2	7.7
Retraction	3	11.5

* Percentage calculated using the total number of non-missing values in each category as the denominator.

**Table 3:** Diagnostic investigation

	n	%*
**Imaging (N = 138)**		
Ultrasound	5	3.6
Mammogram	130	94.2
Not applicable	3	2.2
**Breast imaging reporting and data system score (BIRADS) (N = 135)**		
0 - incomplete	5	3.6
1 - negative	0	0
2 - benign findings	0	0
3 - probably benign	4	2.9
4 - suspicious	46	33.3
5 - highly suspicious of malignancy	80	58.0
6 - known biopsy with proven malignancy	0	0
**Histology type (N = 134)**		
Ductal	120	89.6
Lobular	5	3.7
Sarcoma	2	1.5
Malignant phyllodes	3	2.2
Malignant melanoma	1	0.8
Lymphoma	1	0.8
Squamous carcinoma	1	0.8
Paget’s disease (ductal carcinoma in situ) (DCIS)	1	0.8
**Staging (N = 138)**		
Stage I	15	10.9
Stage II	39	28.3
Stage III	46	33.3
Stage IV	38	27.5

* Percentage calculated using the total number of non-missing values in each category as the denominator.

**Ethical considerations:** the Health Research Ethics Committee of the North-West University approved the study (NWU-00007-19-S1). Approval was also obtained from the North West Provincial Department of Health and the Potchefstroom Hospital Patient's Safety group.

## Results

**Patient demographics:** the demographic characteristics of the study population are described in Table 1. A total of 138 patients (mean age 56.3 (SD: 14.4; 95% CI (53.8-58.7)) had primary invasive breast cancer that was confirmed by histology results, admitted or treated at Potchefstroom Hospital from 1 January 2012 to 31 December 2018. The majority of these patients were female (98.6%), with a mean age of 56.2 (SD: 14.4) (95% CI (54.6-59.7)). One-sample chi-square analysis of age group showed frequency was significantly higher in patients 40 years and above (p < 0.01), peaking in patients between the ages 50 and 59 years, and those above the age of 70 years. Most patients were of African descent (67.4%) or Caucasian (23.9%). A family history of cancer information was only available for 18 patients (13.0%); five patients had a family history of ovarian cancer, compared to four with a family history of uterine and breast cancer, respectively. Only seven patients (5.1%) had a history of prior hormone replacement therapy. About a third of our study population was HIV-positive (27.1%) (Table 1).

The findings of the clinical examination and physical mass description are depicted in Table 2. The majority of patients (51.8%, N = 137) had breast involvements on the left side, 47.5% had right side involvement and 0.7% had bilateral involvement. Lesions occurred mostly in the upper-outer quadrant (43.1%, N = 137), and upper-inner quadrant (20.4%). In 8.8% of patients, the lesion was large enough to involve all four quadrants of the breast, whereas 8.0% of lesions occurred in the retro-areolar area. Tumour size at diagnosis was 2 cm or smaller for 13 patients (9.4%, N = 138), 2 ≤ 5 cm (49.3%), and larger than 5 cm, for 54 (39.1%) of patients. A further three patients had Paget's disease. Tumours mostly had extensions to the skin (25.6%, N = 39), deep structures (15.4%) or both (10.3%). Half of the women aged 30-39 years (N = 20) had tumours 2 ≤ 5 cm, compared to 58.6% of women the 40-49 years' age group (N = 29), 51.6% among women aged 50-59 years (N = 31), and 46.7% of women older than 70 years of age (N = 30). Among women aged 60-69 years (N = 25), the majority (48.0%) presented with tumours larger than 5 cm (data not shown in tables). Other clinical findings included ulcerations in seven patients, discharge (n = 15), skin changes (n = 24), and nipple retractions (n = 2) (Table 2). Table 3 depicts the findings from the analysis of diagnostic investigations. Mammograms were performed for 94.2% (N = 138) of patients (Table 3). About 58% (N = 135) of patients were categorized as BIRADS-5 and 33.33% as BIRADS-4. The most prevalent morphologic pattern was that of ductal carcinoma (89.6%, N = 134) (p < 0.01), whereas five patients (3.7%) presented with lobular carcinoma. Other morphologic patterns indicated were malignant phyllodes tumour (n = 3), malignant melanoma (n = 1), squamous cell carcinoma (n = 1), lymphoma of the breast (n = 1), and Paget's diseases of the breast (n = 3). A one-sample chi-square test performed on the staging of the disease shows that significantly more patients presented in stages II to IV of disease (p < 0.05), with most patients (33.3%, N = 138) presenting at stage III (Table 3).

Compared to ES, LS at time of diagnosis (n = 84) was independent of gender (p = 0.631), age group (p = 0.878), family cancer history (p = 0.381), prior hormone therapy (p = 0.501), HIV status (p = 0.839), population group (p = 0.196), side of involvement (p = 0.058), tumour local invasion (p = 0.301), and histology type (p = 0.950). It was, however, significantly associated with mass location (p = 0.006; Cramér's V = 0.280), tumour size (p < 0.001, Cramér's V = 0.239), and skin changes (p = 0.027, Cramér's V = 0.492). Further analysis within the LS group (N = 84) showed that the majority of patients (33.7%) presented with lesions in the upper-outer quadrant, whereas 19.1% presented with lesions in the upper-inner quadrant. Patients in the LS group mostly presented with tumours larger than 5 cm (50.0%), and the following skin changes: Peau d'orange (10.7%) and puckering (6.0%).

## Discussion

This is the first study describing the diagnostic approach, the epidemiological and clinical profile of breast cancer in Potchefstroom regional hospital, located in a predominantly rural area of South Africa. We showed that most patients in our study consulted at late-stage of the disease, similar to recent studies conducted in peri-urban areas (residential space on the outskirt of large cities) of South Africa [8,13], and other sub-Saharan African countries [14-16]. In contrast, in developed countries such as the United States of America (USA), more than 60% of breast cancer patients present at stages I and II, and only 28% at stage III and III [17]. According to Black and Richmond [14], advanced disease at the time of diagnosis in sub-Sahara African regions may be ascribed to financial, logistical and sociocultural constraints hampering the implementation and sustainability of screening programs. Rambau *et al*. [16] are further of the opinion that this may also be the result of poor referral systems and natural aggressive biological behaviour of tumours.

Our population were mainly from African or Caucasian descent. The distribution of patients could be explained by the local demographics of Potchefstroom municipal area's population group as described in 2011 national statistics, indicating 70.8% as African, 7.1% as Coloured, 1.4% as Asian and 20.6% as Caucasian [5]. Research has, however, shown that the profile of breast cancer varies among different population groups. For example, Vorobiof *et al*. [18] and Walker *et al* . [19], determined that less than 22% of black women in peri-urban areas in South Africa consulted at Stage I and II of breast cancer, with the majority presenting at stage IV. Jedy-Agba *et al*. [20] identified similar trends among black women from the southern African region. In our study, however, although 63.4% and 63.6% of patients from African and Coloured descent, respectively, presented in stages III and IV compared to 51.5% of Caucasian patients, there was no association between population group and staging. Because of the limited sample size and missing data, associations between clinical findings and patient demographic characteristics could not be explored extensively.

The majority of patients in our study population were adults (mean age 56.2 ± 14.4 years (95% CI, 54.6-59.7), which is in agreement with findings from other studies conducted in large urban and peri-urban areas in South Africa [13,21]. Our population was older than the mean age of women in West Africa (mean age for presentation between 35 and 45 years) [22-24], Tanzania (44.7 years) [25], and the Democratic Republic of the Congo (mean of 47.8 ± 12.1 years) [26], although younger than the median age of patients with breast cancer in the USA which is 62 years [27]. This can be ascribed to a higher life expectancy in developed countries than in developing countries [28]. For example, based on population statistics, the highest proportion of the population (both in North West and South Africa) fell within the age category of 0-24 years (with proportions ranging from 9.3% to 11.3%) [5]. Out of the 138 patients, 1.5% (n = 2) were male. This percentage is close to the 1% found by other authors in South Africa [13], and other countries [29-31]. Risk factors are similar in both male and female patients; which include, among others, a family history of cancer (implying a genetic predisposition), radiation exposure history and hormonal imbalances [32]. Because of the small sample size and missing data on family history, it was not possible to contextually investigate this further in our study. Motloutsi *et al*. [33] in a study on male breast cancer at the University of Limpopo, South Africa, affirmed that 97% of participants never heard about breast cancer in males. The authors concluded that the lack of knowledge and awareness regarding male breast cancer among university students may be symptomatic of a more generalised ignorance in the general population [33]. Breast mass was the most constant reason for consultation and finding at clinical examination. In our study, 89.6% of all tumours were invasive ductal carcinoma and 3.7% were invasive lobular carcinoma, which is in line with existing literature indicating that most primary breast cancers are ductal carcinoma, fewer are lobular carcinoma and a smaller number originate from the support tissues (stroma) [34, 35]. There was no large difference on the side affected between left and right (~52% vs. ~48%, respectively), but the tumours were more located in the upper-outer (43.1%) and upper-inner quadrants (20.4%), similar to other previous studies [36-38]. Location of mass was furthermore independent of age, gender, and population group, but did show a statistical and practical significant association with staging. Despite the high likelihood to find breast cancer in the upper-outer quadrant due to the high density and amount of breast tissue, it could not explain the high frequency of breast cancer in a particular quadrant [38]. According to Sohn *et al*. [36], when compared with tumours in other locations, patients with upper-outer quadrant breast cancers have a more favourable survival advantage, whereas high-grade tumours, advanced stage, and race impact survival negatively.

A wide range of extra-mammary tumours can metastasise to the breast. Some common types include carcinoma of the lung, malignant melanoma, serous papillary carcinoma of the ovary and carcinoid tumours [39]. This study presented with only one 34-year old woman with metastatic melanoma, treated for melanoma on the gluteal region a few years prior to the breast mass presentation. Lymphoma of the breast, either as a primary or secondary location, is rare, representing 0.04% to 0.5% of malignant breast tumours [40]. It may also originate from lymphatic tissue present within the breast, adjacent to ducts and lobules, or from intra-mammary lymph nodes [41]. The single patient reported in our distribution was a 24-year old HIV-positive woman with non-Hodgkin's lymphoma of the breast. This is an AIDS-defining cancer and considering the high prevalence of HIV in South Africa, healthcare professionals should always keep it as a differential diagnosis in young patients who are HIV-positive and presenting with breast tumours. Although less than 30% of our population presented with other clinical findings including skin changes, ulcerations, and nipple retractions, these are signs of advanced disease, as per the American Joint Committee on Cancer's (AJCC) Tumor, Node, Metastasis (TNM) breast staging classification and diagnostic features [42]. The latter findings will have an impact on the management and the survival of the patient. The three patients with Paget's disease of the breast, defined by Karakas as a [43] “rare type of cancer of the nipple-areola complex that is often associated with an underlying in situ or invasive carcinoma”, all presented with nipple discharges and peri-areolar eczema-like lesions. These findings are the commonest findings which make even the diagnosis of Paget's disease very suggestive during clinical examination [44, 45]. A total of 130 mammograms were done in the study; eight patients could not have mammography performed because of either the younger age or because of the advanced stage which made it technically impossible to perform. In these patients, ultrasound of the breast was performed as the investigation of choice. The majority of patients had a BIRADS-4 or -5; still, about three percent of patients had BIRADS-3 findings. These findings echoed Lee *et al*. [46] stating that BIRADS-3 is an evolving assessment category. As much as it reduces the number of unnecessary biopsies, it allows the person reading the report to keep a high index of suspicion for detections of early-stage breast cancer. Michaels *et al*. [47] emphasised this by stating that there is significant inter-observer variability in the assessment of mammographic BIRADS-3 findings. Clinicians should, therefore, interpret the BIRADS finding in conjunction with other relevant clinical information.

The HIV-pandemic in South Africa prompted the study to include patients' HIV status. It is important to mention that about a third of the study population was HIV-positive. This percentage is higher than the 19.7% among Soweto women with breast cancer found by Cubasch *et al*. [48], and the 5.3% found in Cape Town, South Africa, by Langenhoven *et al*. [21]. This disparity is probably linked to the prevalence of HIV infection in the general population of these three provinces in South Africa, respectively 22.7% (North West), 17.6% (Gauteng) and 12.6% (Western Cape) [49]. The relationship between HIV/AIDS and breast cancer is still a field of extensive research worldwide. No study has established a direct correlation between the two conditions, but it appears as if these patients are younger with advanced stages of the disease. In the current era of highly active antiretroviral therapy, most of the HIV-positive patients, however, do not experience more complications due to breast cancer than those who are HIV-negative [50]. Finally, our study population comprised of patients from the South African public health sector. This limits the external validity of the study in that results cannot be generalised to the total South African population.

## Conclusion

This study depicts the epidemiological and clinical profile as well as the diagnostic approach of breast cancer in Potchefstroom regional hospital, South Africa. Our study reiterated findings that, irrespective of the geographical status in South Africa, the majority of patients with breast cancer consulted late in both rural and peri-urban areas of developing countries, at stage III or stage IV of the disease, indicating the need for the promotion of breast awareness campaigns, early detection and timeous referral of the patients. Future studies should address aspects of breast cancer management, immunohistochemistry, survival, genetic counselling, and testing in this population.

### What is known about this topic

- Advanced stages of breast cancer at the time of breast cancer diagnosis is associated with poor prognosis;
- Most patients with breast cancer in peri-urban areas in South Africa has been shown to present with advanced disease; however, data on the current status in rural areas are lacking.

### What this study adds

- The epidemiological and clinical profile of breast cancer at the Potchefstroom regional hospital depicts the profile of late-stage breast cancer at the time of diagnosis (60.9%), similar to peri-urban areas in South Africa, and other developing countries;
- Advanced stages of breast cancer at the time of diagnosis was independent of gender, age group, family cancer history, prior hormone therapy, HIV status, population group, side of involvement, tumour local invasion, and histology type.

## Competing interests

The authors declare no competing interests.
